# Assessment and treatment of Down syndrome-associated arthritis: a survey of pediatric rheumatologists

**DOI:** 10.1186/s12969-020-00445-6

**Published:** 2020-07-13

**Authors:** Anna Nicek, Nasreen Talib, Daniel Lovell, Chelsey Smith, Mara L. Becker, Jordan T. Jones

**Affiliations:** 1 Division of General Pediatrics, Children’s Mercy Kansas City, 2401 Gillham Road, Kansas City, MO 64108 USA; 2 Division of Rheumatology, Cincinnati Children’s Hospital Medical Center, 3333 Burnet Avenue, MLC 4010, Cincinnati, OH 45229 USA; 3 Division of Rheumatology, Children’s Mercy Kansas City, 2401 Gillham Road, Kansas City, MO 64108 USA; 4 Division of Rheumatology, Department of Pediatrics, Duke University School of Medicine, 2301 Erwin Road, Durham, NC 27710 USA; 5 University of Missouri-Kansas City, School of Medicine, 2411 Holmes Street, Kansas City, MO 64108 USA

**Keywords:** Pediatric rheumatology, Down syndrome-associated arthritis, Treatment, Diagnosis, Survey

## Abstract

**Background:**

Inflammatory arthritis in children with Down syndrome (DS) was first described in 1984 and is now termed Down syndrome-associated arthritis (DA). Studies have shown that DA is under-recognized with a 19-month average delay in diagnosis. Additionally, most patients present with polyarticular, rheumatoid factor (RF) and anti-nuclear antibody (ANA) negative disease. Current therapies for juvenile idiopathic arthritis (JIA) have been used, but appear to be poorly tolerated, more toxic and less effective in patients with DA. There is currently no standardized approach to the assessment or management of DA. The objective of this study was to describe provider perspectives toward diagnostic and treatment approach of DA, to provide baseline information upon which to design future studies.

**Methods:**

An electronic survey, organized into sections regarding individual practices of assessment and treatment approach of DA, was sent to the Pediatric Rheumatology electronic list-serv. Survey responses were voluntary and results were analyzed by descriptive statistics.

**Results:**

Of 90 survey responses received, 89 were included in the analysis (one was a duplicate response). The respondents were mostly pediatric rheumatologist (94%), with greater than 10 years of experience (55%). The majority (64%) currently see 1–3 patients with DA. Most view DA as the same disease as JIA (73%), and the majority (63%) use a combination of history, exam and imaging to diagnose DA. The most ordered diagnostic tests are CBC (97%) and ESR (96%). The most used treatments include NSAIDs (94%) and methotrexate (91%) followed by anti-TNF agents (90%). Methotrexate is most administered by subcutaneous route (84%) at a dose of 15 mg/m^2^ (56%). Oral corticosteroids were only used in 19% of the patients with DA.

**Conclusion:**

This is the first study to evaluate provider perspectives towards the diagnostic and treatment approach of DA. Most pediatric rheumatologists feel that DA and JIA are synonymous, and similar approaches to diagnosis are employed, utilizing history, physical exam, laboratory tests, and imaging modalities. DA is treated similarly to JIA with initiation of NSAIDs, disease-modifying anti-rheumatic drugs and biologic therapy. More research is needed to determine optimal screening and therapeutic approach specific to DA.

## Background

Down syndrome (DS) is one of the most common birth defects in the United States with approximately 5300 births annually resulting in an estimated birth prevalence of 12.6 per 10,000 live births [1]. DS is a chromosomal disorder characterized by an extensive and heterogeneous phenotype that results from a dosage imbalance of genes located on human chromosome 21 [2]. This results in an increased incidence of oncologic, autoinflammatory and autoimmune conditions that lead to increased morbidity and mortality in those individuals with DS. The comorbidities mentioned include but are not limited to leukemia, celiac disease, thyroid disease, type I diabetes mellitus, and arthritis [3, 4]. Inflammatory arthritis was first described in children with DS in a small case series from 1984 [5]. More recently, children with DS and arthritis have been termed Down syndrome-associated arthritis (DA), and the estimated prevalence of DA has been reported to be 8.7 per 1000 children with Down syndrome [6].

At this time, DA remains under-diagnosed and largely under-reported with an average delay of 19 months from symptom onset to diagnosis [7]. By comparison, the mean time from symptom onset to diagnosis for juvenile idiopathic arthritis (JIA) is 3 months [8, 9]. Adding to the challenges in diagnosis, many patients with DS also present with noninflammatory musculoskeletal abnormalities (i.e. hypotonia, atlantoaxial instability, ligamentous laxity, and pes planus) that can lead to functional limitation [10]. At the time of presentation, most patients with DA have polyarticular disease (five or more joints with active arthritis) that is erosive, and primarily affects the small joints of the hands and wrists [7, 8]. Many patients with DA present to the rheumatology clinic thus therapies utilized to treat JIA often are used to treat DA. The current treatment for JIA is broad and based on many factors, but includes: nonsteroidal anti-inflammatory drugs (NSAIDs), disease-modifying anti-rheumatic drugs (DMARDs), biologic therapies, and glucocorticoids [11]. Most children diagnosed with DA are initially started on NSAID monotherapy and many require a second-line therapy with DMARDs. Of these, the most frequently used is methotrexate despite its risk for toxicity and intolerance [6, 7, 9]. Therapies for JIA have been employed to treat DA with mixed results due to effectiveness and toxicity [7, 12]. Gaps in knowledge related to the optimal therapy and treatment approaches to DA exist currently, and to date, there are no randomized controlled trials or standardized recommendations for the treatment of DA. Many therapeutic agents with different mechanisms of action have been used, but no consistent approach to treatment has been outlined. The aim of this study is to describe the current practices of pediatric rheumatologists, including diagnostic and treatment approaches of DA.

## Methods

Using the REDCap platform, an electronic survey was created which consisted of 12 questions organized into sections regarding responder demographic characteristics, assessment and evaluation, and treatment approach of inflammatory arthritis in Down syndrome. Survey questions used branching logic and asked if providers were aware of DA, how many patients they cared for with DA and how they diagnosed it. Additional questions asked about therapy and sequence of therapies used to treat DA. The survey questions gave multiple choices and, “choose all that apply” questions with a list of laboratory tests, imaging studies, and therapy options. Many questions had an “other” category for the respondent to fill-in responses that may not be listed.

Physicians, including fellows-in-training and nurse practitioners specializing in pediatric, adult, and combined pediatric and adult rheumatology were invited by e-mail to complete the survey. Participants were asked to respond according to their personal experience, not that of institution or group practices or based on medical literature. Respondents were asked to quantify their experience by years of practice and number of cases seen. The survey was electronically sent to the international pediatric rheumatology electronic list-serv (administered by McMaster University, Ontario). All respondents voluntarily completed the survey.

The results were analyzed by descriptive statistics performed using IBM SPSS Statistics version 24.

## Results

Of 90 survey responses, 89 were included in the analyses as one was a duplicate response. The majority (94%) of respondents were pediatric rheumatologists and the remainder were combined adult and pediatric rheumatology providers. Physicians were the respondents in 88 surveys (4 of these were fellow-in training) and one was a nurse practitioner. Over half (55%) had 10+ years of practice experience in the field of rheumatology. Nearly half of the respondents (47%) had greater than 75% time committed to clinical duties, and most practiced in an urban location (94%) and in an academic setting (92%). Many (64%) of those that responded have 1–3 patients with DA that they care for, and 19% have never managed DA in their career. (Table 1).

**Table 1:** Respondent characteristics

Characteristics	n (%)
**Practice Scope**	
Pediatric Rheumatology	84 (94)
Adult Rheumatology	0 (0)
Combined Adult/Pediatric Rheumatology	5 (6)
**Clinic Location**	
Urban	84 (94)
Rural	5 (6)
**Clinic Setting**	
Academic	82 (92)
Private practice	7 (8)
**Experience (by years of practice)**	
0–5 years	24 (27)
5–9 years	16 (18)
10+ years	49 (55)
**Experience (by # of DA**^a^**cases)**	
0	17 (19)
1–3	57 (64)
4–6	10 (11)
7–10	2 (2)
> 10	3 (4)
**Clinical Time**	
0–25%	12 (14)
26–50%	10 (11)
51–75%	25 (28)
> 75%	42 (47)

^a^Down syndrome-associated arthritis

Of the respondents, 73% view the arthritis seen in patients with DS as synonymous with JIA, while 11% viewed it as a separate entity and 16% were unsure. The majority (63%) of respondents diagnosed DA based on a combination of history, exam, and imaging. The most common imaging modalities used were X-ray (55%) and ultrasound (49%). Magnetic resonance imaging (MRI) and computerized tomography (CT) scans were used less frequently at a rate of 27 and 1%, respectively. Most respondents (93%) obtain laboratory tests at diagnosis with complete blood count (CBC) (97%) and erythrocyte sedimentation rate (ESR) (96%) being the most ordered tests. Rheumatoid factor (RF) was the most common arthritis-related laboratory test obtained (82%), followed by human leukocyte antigen B-27 (HLA-B27) (60%). Other laboratory tests including anti-nuclear antigen (ANA), immunoglobulin G (IgG), complement, uric acid, and lactic acid dehydrogenase (LDH) were obtained less frequently (all less than 50%).

**Fig. 1:**
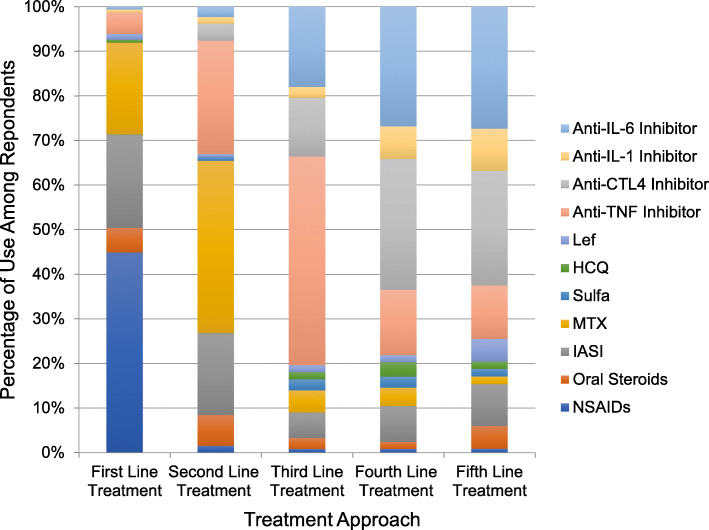
Treatment approach and percentage of use among all respondents. NSAIDs nonsteroidal anti-inflammatory drugs, IASI intra-articular steroid injection, MTX methotrexate, sulfa sulfasalazine, HCQ hydroxychloroquine, Lef leflunomide, Anti-TNF Inhibitor (etanercept, adalimumab, infliximab, golimumab, etc.), Anti-CTL4 (abatacept), Anti-IL-1 (anakinra, canakinumab), Anti-IL-6 (tocilizumab)

Regarding therapeutic treatment approaches, 74% used NSAIDs as first line-treatment. The survey then asked for subsequent treatment choices after therapeutic failure. For those that failed first-line therapy, methotrexate was most often used as second-line treatment at 56%. TNF-inhibitors were most frequently used as a third-line agent at 64%. Anti-CTLA4 agents were most frequently used as fourth line therapy, and anti-IL-6 inhibitors were the most common fifth-line therapy in treatment when others had failed. IASI (intra-articular steroid injections) were used in small percentages during all treatment attempts, but oral steroids were used in small percentages during the first and second attempts only (Fig. 1). Methotrexate was more commonly given via subcutaneous route compared to the oral formulation and was more commonly dosed at 15 mg/m2 once weekly compared to < 15 mg/m2 once weekly. The survey specifically asked about which therapies providers avoid in children with DA and asked that respondents provide an explanation as to why they were avoided. Oral steroids were avoided most frequently (19%) and comments provided by participants indicate that steroids were mostly avoided due to the adverse effects such as weight gain, risk for gastrointestinal bleed, risk for infection, mood changes, and exacerbation of comorbid conditions such as insulin dependent diabetes mellitus. Leflunomide was avoided in 17% and methotrexate was avoided in 7% due to concern for intolerance, cytopenias and liver toxicity associated with both medications. Leflunomide was specifically avoided due to the lack of an oral suspension and the long half-life of the drug. Respondents also avoided Plaquenil (15%) and sulfasalazine (14%) with most comments indicating concern for adverse effects and lack of efficacy of those therapies in treating arthritis. There were 5% that avoided anti-TNF agents, mostly due to concern for increased risk of malignancy, and 3% that avoided anti-IL-6 inhibitors due to concern for cytopenias.

## Discussion

Down syndrome-associated arthritis (DA) remains a significant source of morbidity for children with Down syndrome (DS) [6]. Previous studies have shown that DA is under-recognized with delays in diagnosis, frequently presents with extensive joint involvement, and optimal treatment approach and escalation remains unclear [6–8]. This survey describes the real-world approach to diagnosis and treatment of DA by surveyed pediatric rheumatologists.

Although most respondents felt that arthritis in a patient with DS is the same as JIA, we would argue that these may in fact be different diseases despite a similar phenotypic presentation. As JIA is idiopathic in nature, for it to occur in a child with DS may contradict that idea, as the arthritis may be due to downstream metabolic and immunologic effects from the genetic abnormality of DS. In a cross-sectional survey of parents to children with DS in New York a prevalence of arthritis was reported to be 0.2% [13], which would indicate arthritis is uncommon in DS. Additionally, 19% of respondents to this survey have never managed a case of DA, however, newer studies suggest the incidence and prevalence of DA are nearly 2–3 times greater than previously thought [7, 8, 14]. This is thought to be due to lack of awareness of arthritis in DS and features of DS including joint hypermobility, intellectual disability, and poor language skills that may make diagnosis more difficult [8]. More recent studies are beginning to find distinct differences between DA and JIA such as lack of elevated inflammatory markers and ANA positivity in DA compared with JIA [7, 8]. Additionally, more reports suggest that trisomy 21 promotes pro-inflammatory cytokines and T cell dysregulation that results in chronic autoinflammation and immune dysregulation that could be the driver of DA and other autoinflammatory diseases seen in children with DS [15–17].

As the rheumatologic and immunologic community is becoming more aware of these findings, more focused efforts have been put forth to explore differences between the two diseases. A recent study in children with DA did show immunologic differences between DA and JIA, with those with DA having increased polyreactive T-helper cells, more elevation of serum inflammatory cytokines and reduced T-regulatory cells, as well as a marked increase in synovial inflammation compared to JIA [16]. While this may indicate a different pathogenesis for DA compared with JIA, more evidence is needed to clearly elucidate and fully explore the differences. Additionally, previous reports indicate subtle clinical differences between DA and JIA such as the primary presentation and response to therapy, which may be attributed to the heterogeneous nature of JIA or rather a different pathogenesis in DA entirely. Further sophisticated immunologic studies are needed to determine differences, if any, between JIA and DA. Additionally, a relative lack of exposure to or experience treating DA may explain the reason most pediatric rheumatologists feel that JIA and DA are the same disease, as many of the rheumatologists surveyed had only managed 1–3 cases of DA and nearly 20% had never managed DA at all. Part of the discrepancy observed in these responses may be owed to practice type (size, academic affiliation), location (city, region), and years of practice. However, the correlation between these factors and the clinical experience with DA was not assessed in this survey as our focus was mainly to identify diagnosis and management approaches of DA among pediatric rheumatology providers.

Furthermore, there is likely a lack of awareness of DA among not only rheumatologists, but also the general public and primary care providers. In the most recent version of the American Academy of Pediatrics Health Supervision of Children with Down Syndrome [18] used by physicians to help guide screening in patients with DS there is no mention of surveillance for arthritis. Not addressed in our study was the reason for referral to rheumatologists surveyed. This would be interesting to further investigate where children were initially evaluated for symptoms and what prompted referral to a rheumatologist. Assessment for screening and diagnostic criteria among primary care physicians would be helpful in recognizing potential areas for education regarding arthritis in the DS population.

The diagnosis of JIA is a clinical diagnosis based on the Edmonton 2001 International League of Associations for Rheumatology (ILAR) criteria [19], however, there is no clear guidance or criteria for the diagnosis of DA. We found that clinicians surveyed use a similar diagnostic approach to evaluate for DA as they would JIA by ordering more commonly used tests such as CBC, CRP, ESR and RF, which could be explained if both diseases are considered synonymous. However, this approach may be problematic, as studies have shown that up to 81% of children with DA present with polyarticular-RF-negative arthritis, a proportion significantly greater compared to 19% of children with JIA [8, 20]. As well, studies have found that CRP and ESR are elevated significantly less frequently in DA compared to JIA [7, 8]. Respondents similarly use radiologic modalities to help guide diagnosis of DA. X-ray was most frequently used, likely as it is readily accessible, reproducible, and relatively inexpensive. Unfortunately, X-ray imaging, despite its relative ease is not sensitive enough to reveal subtle or early arthritis, thus when bony changes are present on X-ray, it may indicate long term involvement and irreversible joint damage. Ultrasound was the second most used imaging modality as it has similar accessibility, is easily tolerated, and has the added benefit of avoiding radiation exposure as compared to X-rays. A limitation is that it is highly operator dependent and may not be as reproducible from one technician to another, making it more difficult to follow disease involvement over time [21]. Another limitation is the lack of validated standards for assessing the implications of ultrasound findings in JIA and a complete lack of ultrasound studies in DA [21]. Fewer respondents reported MRI use, which is likely related to the high cost, limited availability, and risk of complications for children with DS requiring sedation [8]. Previous studies have shown that up to 80% of children with DA will present with erosive disease and extensive joint changes noted on X-ray at presentation [7]. Our study showed that providers are frequently using imaging modalities to help diagnosis, and we feel it would be reasonable to encourage the use of imaging early in the diagnostic workup if suspicion for DA is high. The idea of using ultrasound as part of a novel screening tool to potentially diagnose DA sooner and help follow disease is an intriguing idea but would need thorough validation in this population of patients.

Our study found that rheumatologists use treatment strategies for DA that include initiation of NSAIDs, localized steroid injections, DMARDs, and biologics. NSAIDs and intra-articular steroid injections (IASI) were used most often as first line treatment of DA, which is likely due to their relatively low side effect profile. IASI can be quickly performed in the office if the child is easily distractible and compliant, however, the child may require sedation for the procedure, and appropriate measures should be taken to ensure safe sedation for a child with DS [22]. Beyond first line treatment with NSAIDs and IASI, most initiated methotrexate at the JIA maximally effective recommended dose of 15 mg/m^2^ body surface area once weekly despite the known increase in toxicity of methotrexate in children with DS [23]. Biologic therapies, most often anti-TNF and CTLA4 agents, were used as third and fourth line treatments following the JIA guideline recommendation to initiate biologic therapies for more severe, DMARD-resistant and/or polyarticular disease [11]. Oral corticosteroids were the treatment that respondents most frequently listed as being avoided. This appears to be due to the side effect profile, issues related to weight gain, obesity, adverse effects, and comorbid issues associated with DS [24].

Common adverse effects of methotrexate include gastrointestinal toxicities (mucositis), liver toxicity, and myelosuppression [25]. It is known that children with DS getting treatment with high dose methotrexate for leukemia as well as those getting low dose methotrexate for the treatment of arthritis often require frequent lab monitoring and dose adjustment as a result of those side effects [7]. This is thought in part, to be due to gene dosage effects for the multiple folate pathway enzymes encoded on chromosome 21 [12]. For example, increased intracellular transport of methotrexate via SLC19A1 and higher cellular methotrexate polyglutamates have been documented in hyperdiploid acute lymphoblastic leukemia cells with extra copies of chromosome 21 [26]. Nonetheless, our results show that many providers still use methotrexate. This may be due to the efficacy of the medication to treat arthritis, despite the potential for adverse effects and toxicity. However, there is also a lack of evidence to suggest the best method to treat DA and the best strategy to escalate therapy. There have been no studies to date that have evaluated the best treatment practices for children with DA, and since this was a study of rheumatologists, this likely explains why most providers have followed therapy guidelines for JIA. Further studies are needed to identify safe and efficacious therapies for DA as well as ideal escalation of therapy.

Our study has several limitations including the low number of respondents, however, most respondents felt JIA and DA were the same disease, and additionally this was reflected in the assessment and treatment responses, which would likely result in similar findings even with a larger number of respondents. Participation bias was another limitation to our study as it is possible that many chose not to complete the survey due to lack of experience with DA, however, an increase in respondents with only a few or no cases seen may not significantly alter the results. Another limitation is that respondents may have answered the survey questions based on information other than their personal experience due to the hypothetical nature of the questions and lack of details that would be present in a case-based survey. However, the focus of this survey was intended to describe current assessment and treatment approach to develop a baseline for evaluation and therapy.

## Conclusion

This is the first study to evaluate the current practices of diagnostic and treatment approaches of DA. Our study found that most pediatric rheumatologists feel that DA and JIA are synonymous and thus a similar approach to diagnosis is employed, utilizing history, physical exam, laboratory tests, and imaging. We also found that the treatment approach to DA is like JIA, which is likely due to lack of treatment guidelines for patients with DA. More studies are needed to determine optimal screening and therapeutic approaches for patients with DA.

## Data Availability

The datasets used and analyzed during the current study are available from the corresponding author on reasonable request.

## Abbreviations

DS: Down syndrome
RF: Rheumatoid factor
ANA: Anti-nuclear antibody
JIA: Juvenile idiopathic arthritis
DA: Down syndrome-associated arthritis
NSAIDs: Nonsteroidal anti-inflammatory drugs
DMARDs: Disease-modifying anti-rheumatic drugs
MRI: Magnetic resonance imaging
CT: Computerized tomography
HLA-B27: Human leukocyte antigen B-27
IgG: Immunoglobulin G
LDH: Lactic acid dehydrogenase
IASI: intra-articular steroid injections
ILAR: International League of Associations for Rheumatology
